# Health-related quality of life after traumatic brain injury: deriving value sets for the QOLIBRI-OS for Italy, The Netherlands and The United Kingdom

**DOI:** 10.1007/s11136-020-02583-6

**Published:** 2020-07-15

**Authors:** Daphne C. Voormolen, Suzanne Polinder, Nicole von Steinbuechel, Yan Feng, Lindsay Wilson, Mark Oppe, Juanita A. Haagsma, the CENTER-TBI participants and investigators, Cecilia Åkerlund, Hadie Adams, Krisztina Amrein, Nada Andelic, Lasse Andreassen, Audny Anke, Anna Antoni, Gérard Audibert, Philippe Azouvi, Maria Luisa Azzolini, Ronald Bartels, Pál Barzó, Romuald Beauvais, Ronny Beer, Bo-Michael Bellander, Antonio Belli, Habib Benali, Maurizio Berardino, Luigi Beretta, Morten Blaabjerg, Peter Bragge, Alexandra Brazinova, Vibeke Brinck, Joanne Brooker, Camilla Brorsson, Andras Buki, Monika Bullinger, Manuel Cabeleira, Alessio Caccioppola, Emiliana Calappi, Maria Rosa Calvi, Peter Cameron, Guillermo Carbayo Lozano, Marco Carbonara, Ana M. Castaño-León, Giorgio Chevallard, Arturo Chieregato, Giuseppe Citerio, Maryse Cnossen, Mark Coburn, Jonathan Coles, Jamie D. Cooper, Marta Correia, Amra Čović, Nicola Curry, Endre Czeiter, Marek Czosnyka, Claire Dahyot-Fizelier, Helen Dawes, Véronique De Keyser, Vincent Degos, Francesco Della Corte, Hugo den Boogert, Bart Depreitere, Đula Đilvesi, Abhishek Dixit, Emma Donoghue, Jens Dreier, Guy-Loup Dulière, Ari Ercole, Patrick Esser, Erzsébet Ezer, Martin Fabricius, Valery L. Feigin, Kelly Foks, Shirin Frisvold, Alex Furmanov, Pablo Gagliardo, Damien Galanaud, Dashiell Gantner, Guoyi Gao, Pradeep George, Alexandre Ghuysen, Lelde Giga, Ben Glocker, Jagoš Golubovic, Pedro A. Gomez, Johannes Gratz, Benjamin Gravesteijn, Francesca Grossi, Russell L.Gruen, Deepak Gupta, Juanita A. Haagsma, Iain Haitsma, Raimund Helbok, Eirik Helseth, Lindsay Horton, Jilske Huijben, Peter J. Hutchinson, Bram Jacobs, Stefan Jankowski, Mike Jarrett, Ji-yao Jiang, Kelly Jones, Mladen Karan–, Angelos G. Kolias, Erwin Kompanje, Daniel Kondziella, Lars-Owe Koskinen, Noémi Kovács, Alfonso Lagares, Linda Lanyon, Steven Laureys, Fiona Lecky, Rolf Lefering, Valerie Legrand, Aurelie Lejeune, Leon Levi, Roger Lightfoot, Hester Lingsma, Andrew I. R. Maas, Marc Maegele, Marek Majdan, Alex Manara, Geoffrey Manley, Costanza Martino, Hugues Maréchal, Julia Mattern, Charles McFadyen, Catherine McMahon, Béla Melegh, David Menon, Tomas Menovsky, Davide Mulazzi, Visakh Muraleedharan, Lynnette Murray, Nandesh Nair, Ancuta Negru, David Nelson, Virginia Newcombe, Daan Nieboer, Quentin Noirhomme, József Nyirádi, Otesile Olubukola, Matej Oresic, Fabrizio Ortolano, Aarno Palotie, Paul M. Parizel, Jean-François Payen, Natascha Perera, Vincent Perlbarg, Paolo Persona, Wilco Peul, Anna Piippo-Karjalainen, Matti Pirinen, Horia Ples, Suzanne Polinder, Inigo Pomposo, Jussi P. Posti, Louis Puybasset, Andreea Radoi, Arminas Ragauskas, Rahul Raj, Malinka Rambadagalla, Ruben Real, Jonathan Rhodes, Sylvia Richardson, Sophie Richter, Samuli Ripatti, Saulius Rocka, Cecilie Roe, Olav Roise, Jonathan Rosand, Jeffrey V. Rosenfeld, Christina Rosenlund, Guy Rosenthal, Rolf Rossaint, Sandra Rossi, Daniel Rueckert, Martin Rusnák, Juan Sahuquillo, Oliver Sakowitz, Renan Sanchez-Porras, Janos Sandor, Nadine Schäfer, Silke Schmidt, Herbert Schoechl, Guus Schoonman, Rico Frederik Schou, Elisabeth Schwendenwein, Charlie Sewalt, Toril Skandsen, Peter Smielewski, Abayomi Sorinola, Emmanuel Stamatakis, Simon Stanworth, Ana Stevanovic, Robert Stevens, William Stewart, Ewout W. Steyerberg, Nino Stocchetti, Nina Sundström, Anneliese Synnot, Riikka Takala, Viktória Tamás, Tomas Tamosuitis, Mark Steven Taylor, Braden Te Ao, Olli Tenovuo, Alice Theadom, Matt Thomas, Dick Tibboel, Marjolein Timmers, Christos Tolias, Tony Trapani, Cristina Maria Tudora, Peter Vajkoczy, Shirley Vallance, Egils Valeinis, Zoltán Vámos, Gregory Van der Steen, Joukje van der Naalt, Jeroen T. J. M. van Dijck, Thomas van Essen, Wim Van Hecke, Caroline van Heugten, Dominique Van Praag, Thijs Vande Vyvere, Audrey Vanhaudenhuyse, Roel P. J. van Wijk, Alessia Vargiolu, Emmanuel Vega, Kimberley Velt, Jan Verheyden, Paul M. Vespa, Anne Vik, Rimantas Vilcinis, Victor Volovici, Nicole von Steinbüchel, Daphne Voormolen, Petar Vulekovic, Kevin K. W. Wang, Eveline Wiegers, Guy Williams, Lindsay Wilson, Stefan Wolf, Zhihui Yang, Peter Ylén, Alexander Younsi, Frederik A. Zeiler, Veronika Zelinkova, Agate Ziverte, Tommaso Zoerle

**Affiliations:** 1 Department of Public Health, Erasmus MC, University Medical Center Rotterdam, PO Box 2040, 3000 CA Rotterdam, The Netherlands; 2 Institute of Medical Psychology and Medical Sociology, Georg-August-University, Waldweg 37, 37073 Göttingen, Germany; 3 Centre for Primary Care and Public Health, Queen Mary University of London, London, UK; 4 Department of Psychology, University of Stirling, Stirling, UK; 5 Axentiva Solutions, C/Calvario, 271-B 1º IZQ, Tacoronte, 38350 Santa Cruz de Tenerife, Spain; 6 Department of Emergency Medicine, Erasmus MC, University Medical Center Rotterdam, Rotterdam, The Netherlands

**Keywords:** Health-related quality of life, Quality of life after brain injury overall scale (QOLIBRI-OS), Health utilities, Value set, Traumatic brain injury

## Abstract

**Purpose:**

The Quality of Life after Brain Injury overall scale (QOLIBRI-OS) measures health-related quality of life (HRQoL) after traumatic brain injury (TBI). The aim of this study was to derive value sets for the QOLIBRI-OS in three European countries, which will allow calculation of utility scores for TBI health states.

**Methods:**

A QOLIBRI-OS value set was derived by using discrete choice experiments (DCEs) and visual analogue scales (VAS) in general population samples from the Netherlands, United Kingdom and Italy. A three-stage procedure was used: (1) A selection of health states, covering the entire spectrum of severity, was defined; (2) General population samples performed the health state valuation task using a web-based survey with three VAS questions and an at random selection of sixteen DCEs; (3) DCEs were analysed using a conditional logistic regression and were then anchored on the VAS data. Utility scores for QOLIBRI-OS health states were generated resulting in estimates for all potential health states.

**Results:**

The questionnaire was completed by 13,623 respondents. The biggest weight increase for all attributes is seen from “slightly” to “not at all satisfied”, resulting in the largest impact on HRQoL. “Not at all satisfied with how brain is working” should receive the greatest weight in utility calculations in all three countries.

**Conclusion:**

By transforming the QOLIBRI-OS into utility scores, we enabled the application in economic evaluations and in summary measures of population health, which may be used to inform decision-makers on the best interventions and strategies for TBI patients.

**Electronic supplementary material:**

The online version of this article (10.1007/s11136-020-02583-6) contains supplementary material, which is available to authorized users.

## Introduction

Traumatic Brain Injury (TBI) is generally defined as “an alteration in brain function or other evidence of brain pathology, caused by an external force” [1]. TBI is one of the leading causes of death and disability worldwide [2]. Annually, TBI costs approximately $US 400 billion worldwide and imposes a massive burden on society [3]. Economic evaluations in health care interventions are increasingly being used to inform governments, healthcare funders and policy makers and to prioritize resource allocation [4]. Nonetheless, for economic evaluations, preference-based measures (PBMs) are a requirement [5] and values have to be assigned to the health states these measures describe [6]. Many popular PBMs are generic. However, generic instruments do not always adequately assess specific aspects of health-related quality of life (HRQoL) that are affected by a disease such as cognition [7]. Therefore, generic measures, such as the EuroQol-5D (EQ-5D) and Short Form-36 (SF-36), are often combined with condition-specific questionnaires. A TBI-specific instrument is the Quality of Life after Brain Injury overall scale (QOLIBRI-OS) [8]. The QOLIBRI-OS instrument is a disease-specific tool for assessing HRQoL after sustaining TBI, which covers areas that are typically affected by TBI [9]. It was developed in 2012 and since then has been widely applied in TBI [8]. By generating a condition-specific preference-based measure (CSPBM) for TBI, it will potentially provide a more accurate assessment of the impact of heterogeneous outcomes after TBI and a more sensitive measure of the benefit of interventions.

The QOLIBRI-OS is a non-preference-based instrument that yields ordinal data, and therefore has limitations for economic evaluation studies. Transforming QOLIBRI-OS into utility scores will enable the application in economic evaluations and in summary measures of population health (e.g. quality-adjusted life years (QALYs)). Furthermore, a value set for the QOLIBRI-OS will introduce the ability to summarize general population preferences for health states that could be experienced by TBI patients and the HRQoL of TBI patients can be compared with other (patient) groups.

To be able to use health state values in QALYs calculations [10], they have to be anchored on a scale from 0 (dead) to 1 (full health). A less than 0 value is given to health states which are reported to be worse than dead. Ultimately, a value set can be generated, which means that each item level of the QOLIBRI-OS has a weight (utility) assigned to it. A lower utility means a higher impact on HRQoL. Each QOLIBRI-OS health state can be converted into a single summary index value with a value set.

Value sets for generic instruments (e.g. EQ-5D and Health Utility Index 2 (HUI2)) [11] are widely available and are being used extensively in economic evaluations [12]. However, the QOLIRBI-OS currently does not have utilities, which means the instrument cannot be used for QALY calculations [13]. To make the QOLIBRI-OS suitable for use in economic evaluations, the health states need to be valued with a preference-elicitation method. Widely used methods are discrete choice experiments (DCE) [14, 15] and visual analogue scales (VAS) [16]. The DCE and VAS technique are used to quantify health outcomes [17–21]. DCEs are based on stated preferences and are seen to be a simpler method than the conventional methods such as time trade off (TTO) and standard gamble (SG) [22]. The DCE approach makes it possible to predict values for alternatives in hypothetical situations or conditions that cannot be judged in the real world [23]. The VAS is a valuation technique that records participants’ views about hypothetical health states on a scale from 0 (worst imaginable health state) to 100 (best imaginable health state) [16].

The objective of this study was to develop health utility indices for the QOLIBRI-OS health states. In order to do this, we aimed to develop value sets for the QOLIBRI-OS in three European countries by the use of a web-based DCE and VAS valuation study, which will allow calculation of utility values for the health states measured with the QOLIBRI-OS.

## Methods

The QOLIBRI-OS is a short, six-item version of the Quality of Life after Brain Injury (QOLIBRI), which provides a profile of HRQoL in domains typically affected by brain injury. It addresses well-being and functioning and the psychometric properties have been determined satisfactory to good [8]. The QOLIBRI-OS assesses a single overall score, which provides a brief summary measure of HRQoL [8]. The six items are satisfaction with physical condition; how brain is working, in terms of concentration, memory and thinking; feelings and emotions; ability to carry out day to day activities; personal and social life; current situation and future prospects. Responses are on a 5-point Likert scale ranging from “not at all satisfied” to “very satisfied”. Ultimately, the current situation and future prospects item from the QOLIBRI-OS was excluded because a general sample might answer this item too subjectively, which may hamper the use of the QOLIBRI-OS value set in populations other than TBI patients. By use of Rasch analysis, the psychometric validity of the QOLIBIR-OS scale was tested and well-functioning items of the QOLIBRI-OS were identified, which ultimately resulted in measures of item difficulty and fit of the QOLIBRI-OS. The scale was examined for redundancy and removing the current situation and future prospects item did not change the properties of the scale (Online Appendix A). In the end, the QOLIBRI-OS scale included 5 items, each with 5 levels, which are shown in Table 1.

**Table 1:** Five selected items of QOLIBRI-OS

QOLIBRI-OS
1. Satisfied with physical condition
2. Satisfied with how brain is working, in terms of concentration, memory and thinking
3. Satisfied with feelings and emotions
4. Satisfied with ability to carry out day to day activities
5. Satisfied with personal and social life

Item levels: 1-Not at all; 2-Slightly; 3-Moderately; 4-Quite; 5-Very

**Fig. 1:**
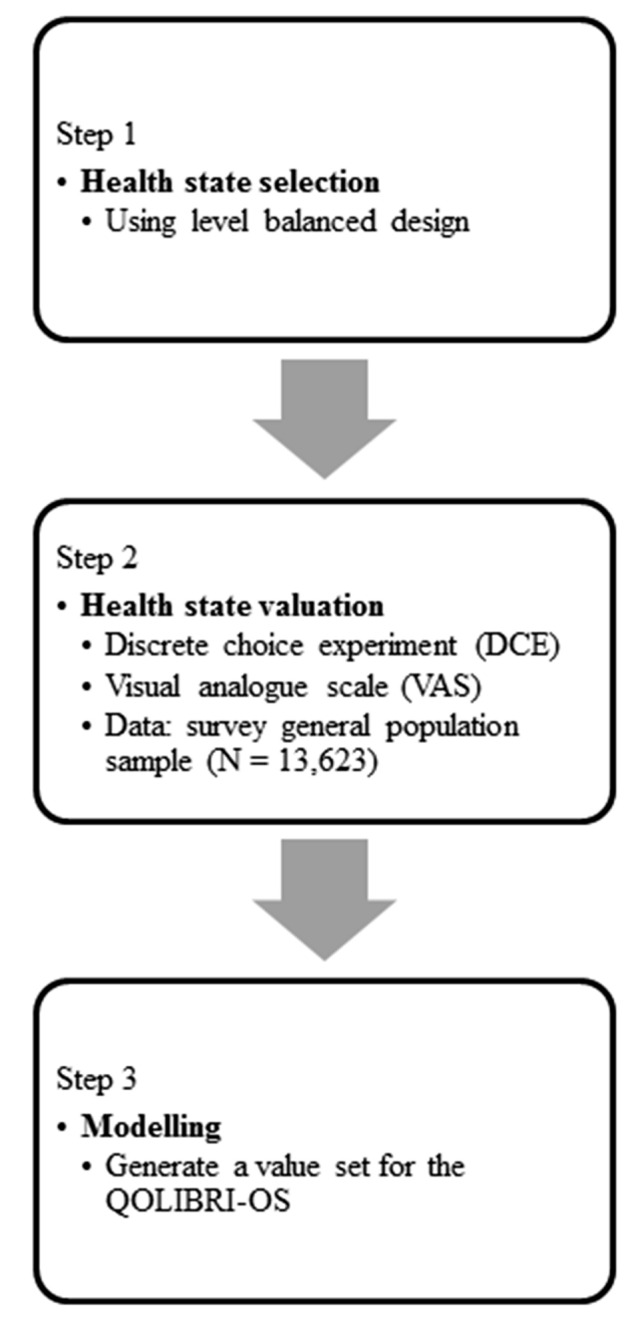
Steps taken to yield a QOLIBRI-OS value set which enables calculation of utility weights for the health states measured with this instrument. *QOLIBRI* Quality of Life after Brain Injury, *QOLIBRI-OS* Quality of Life after Brain Injury Overall Scale

Developing value sets for the QOLIBRI-OS required three methodological steps (Fig. 1). Each of these steps is described in more detail in the following sections.

### Step-by-step

#### Step 1: Health state selection

Even after reducing the items from the QOLIBRI-OS from 6 to 5, the selected items can generate a large number of possible health states. The 5-item QOLIBRI-OS can generate 3125 (5^5^) possible health states, since each dimension has five levels, and this makes it impossible to ask the respondents for a valuation all of them [13]. We therefore made a selection of health states to be used in the health state valuation task. For the DCE valuation of the QOLIBRI-OS, 392 health states were selected, which were presented in 196 pairs, based on a method devised by Oppe and van Hout [24]. These health states cover the spectrum of severity. For this we used a level-balanced design [13], meaning that all levels of each item occurred with the same frequency. The same 392 states were used in the EuroQol EQ-5D-5L value set valuation study [24–26]. The best and worst health states plus death were selected for the visual analogue scale (VAS) valuation.

#### Step 2: Health state valuation—study design

During this step a panel of judges evaluated the selected health states. The general population was asked to evaluate the possible QOLIBRI-OS health states by assuming what they would consider their quality of life to be, if they were in one of these specific health states. The responses from the general population sample were used to generate the health state values.

### Health state valuation—Survey

The web-based questionnaire included questions regarding the demographics of the respondent (e.g. age, sex, educational and income level, chronic health complaints), sixteen DCE questions and three VAS questions. The DCE pairs were randomly assigned to the participants. During this study, no DCE or VAS data were excluded. The survey and description of health states were translated from English into Dutch and Italian using translation software and subsequently translated back into English. Bilingual native speakers verified the translations independently. The panel of judges consisted of members of the general public aged 18 to 75 years from the United Kingdom (UK), Italy and the Netherlands, which provided an international spread. The samples were also representative of the population in the countries with regard to age, gender and education. A total number of 13,623 respondents filled out the questionnaire (Italy: 5270 respondents; Netherlands 4183 respondents; UK 4170 respondents). The questionnaires were distributed by a market research agency (Survey Sampling International (SSI), nowadays called Dynata) via internet during the period 29 June till 31 July 2017. A second round of data collection took place between 3 February and 16 February 2018 to collect some more responses for the VAS data, especially for the health state ‘dead’, and these were all respondents who had already completed the survey the first round (recontacts).

### Valuation techniques

The responses from the general population sample reflect preferences between different health states [10] and these were used to generate and model the value sets.

**Fig. 2:**
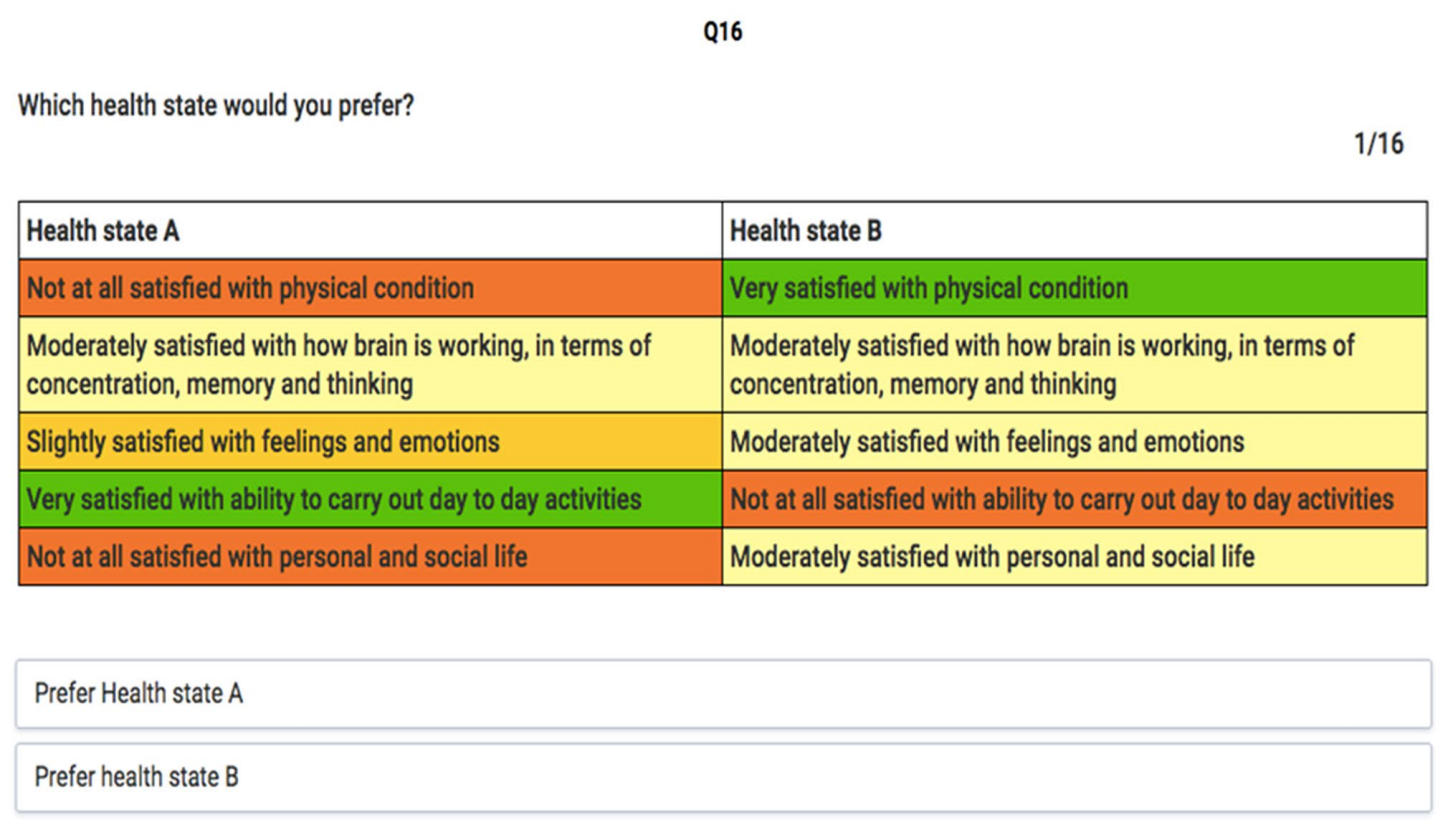
Example of a QOLIBRI-OS DCE question. *DCE* Discrete Choice Experiment

One of the methods used to evaluate the health states was a DCE [27–29], which is an ordinal measurement method. With this method, a pair of health states (labelled A and B, Fig. 2), with no reference to the duration of the states, is presented, and respondents have to decide which health state they consider to be better. No indifference option was included. The assumption of a DCE is that the choices among sets of divergent health states are driven by differences in the levels of the dimensions from the QOLIBRI-OS which define the health states. Furthermore, by asking respondents to choose between health states with altering severity levels and different combinations, the opportunity arises to estimate the impact of the preferences based on the changes in levels [30]. We used colours in the online survey to indicate the severity level of the attribute, ranging from green indicating very satisfied to red indicating not at all satisfied.

The second method used was the VAS, which is a valuation technique that requires participants to score the injury stage on a vertical scale graded from 0 (worst imaginable health state) to 100 (best imaginable health state). As done previously by Stolk et al. [23], a rescaled VAS, based on dead and the best and worst health states, was developed; health preference valuations of 0 to 100 on the VAS were rescaled from 0 to 1. This was done by the use of the following formula:

$${\text{VAS rescaled}} = 100 \times \frac{VAS\,mean - VAS\,dead}{{VAS\,11,111 - VAS\,dead}}.$$

It was necessary to rescale the VAS values in such a way that the value for death was explicitly set at 0 and the best health state (11,111) to 1 [23].

#### Step 3. Modelling the DCE health state valuations

Statistical modelling was used to estimate the values for all potential health states according to the responses for the selected health states. The coefficients for each level and attribute were estimated by regression techniques. Whether a level has a positive or negative effect on utility depends on the sign of the coefficient. The relative importance of the level is revealed by the value of the coefficient. A level is considered to be important when the coefficient has been determined to be statistically significant (*p* < 0.05) [31]. Afterwards, the values for all the health states described by the QOLIBRI-OS were generated from these coefficients [32]. A utility score for the QOLIBRI-OS health states is generated from the DCE responses anchored on the VAS. DCE responses were defined as binary outcomes.

As described by Feng et al. [32], a 20 parameter model (4 levels × 5 dimensions = 20 parameters) was generated for the QOLIBRI-OS, which estimated four parameters for each dimension and one parameter per level, with the “very satisfied” level used as the reference. This model allowed for the coefficients to differ between dimensions and for the importance of each level of problems to differ between dimensions [32]. Regression models were estimated for the DCE in all three countries separately. DCE answers were analysed using a conditional logistic regression. Subsequently, we derived health state values from the DCE data on the QALY scale by anchoring the values on the estimated VAS value for the worst state (55,555). The following formula was used for this process:

$${ }\beta_{anchored\,DCE\,model } = \frac{{\beta_{20\,parameter\,DCE\,model} \times estimated\,VAS_{worst\,state} }}{{estimated\,DCE_{worst\,state} }},$$

where $\beta_{20\,parameter\,DCE\,model}$ is the coefficient from the conditional logistic regression DCE model, $estimated\,VAS_{worst\,state}$ is the pooled mean value given to the worst health state by all respondents, $estimated\,DCE_{worst\,state}$ is the intercept and all “not at all” level coefficients from the DCE model summed up, which generates a $\beta_{anchored\,DCE\,model }$ for each attribute and level as output. The utilities are based on and have been calculated by the use of these anchored DCE coefficients.

In addition, we implemented a generalized additive logistic regression using the bamlss package of R [33, 34] to relax the assumption of the standard logistic regression on the linear relationship between the predictors and the log-odds of the outcome. We compared the non-parametric model specifying an additive (but otherwise unknown) utility function to the standard model assuming linear utility.

### Statistical analysis

For the statistical analyses, responses on the QOLIBRI-OS were recoded with 1 reflecting “very satisfied” and 5 reflecting “not at all satisfied” (similar to the convention used for the EQ-5D). Therefore, 11,111 was seen as the ‘best health state’ and 55,555 as the ‘worst health state’.

Rasch analysis was performed using Winsteps 3.92 (Winsteps.com, Chicago Illinois, USA).

All other analyses were performed using SPSS version 24 for Windows (IBM SPSS Statistics, SPSS Inc, Chicago, IL) and R (R Core Team (2013). R: A language and environment for statistical computing. R Foundation for Statistical Computing, Vienna, Austria).

## Results

### Study population

The characteristics of the survey respondents are shown in Table 2. A total of 13,623 respondents divided over three countries completed the questionnaire. The median age of the respondents was 45 years old. Approximately half of the respondents (51.2%; *N* = 6981) were employed and 15% (*N* = 2068) were retired. One out of two respondents have experienced serious illness in their immediate family and/or reported to have chronic health complaints.

**Table 2:** Characteristics of the study population

	All respondents	UK	The Netherlands	Italy
	(*N* = 13,623)	(*N* = 5270)	(*N* = 4183)	(*N* = 4170)
	*N* (%)	*N* (%)	*N* (%)	*N* (%)
Age^a^ (years)	45 [33–57]	44 [32–57]	46 [33–58]	45 [34–57]
Gender (male)	6736 (49.4%)	2597 (49.3%)	2069 (49.5%)	2070 (49.6%)
Education^b^				
Low	3797 (27.9%)	1205 (22.9%)	1232 (29.5%)	1360 (32.6%)
Middle	6499 (47.7%)	2265 (43.0%)	1901 (45.4%)	2333 (55.9%)
High	3327 (24.4%)	1800 (34.2%)	1050 (25.1%)	477 (11.4%)
Work status^c^				
Employed	6981 (51.2%)	2759 (52.4%)	2214 (52.9%)	2008 (48.2%)
Unemployed	1915 (14.1%)	475 (9.0%)	447 (10.7%)	993 (23.8%)
Looking after others^d^	699 (5.1%)	358 (6.8%)	177 (4.2%)	164 (3.9%)
Student	849 (6.2%)	294 (5.6%)	270 (6.5%)	285 (6.8%)
Retired	2068 (15.2%)	855 (16.2%)	545 (13.0%)	668 (16.0%)
Unable to work	1111 (8.2%)	529 (10.0%)	530 (12.7%)	52 (1.2%)
Annual household income^e^				
Low	3131 (23.0%)	1126 (21.4%)	759 (18.1%)	1247 (29.9%)
Middle	3315 (24.3%)	1604 (30.4%)	728 (17.4%)	983 (23.6%)
High	5076 (37.3%)	1994 (37.8%)	1787 (42.7%)	1295 (31.1%)
Do not know/do not want to tell	2100 (15.4%)	546 (10.4%)	909 (21.7%)	645 (15.5%)
Experience of serious illness				
In you yourself (yes)	3517 (25.8%)	1834 (34.8%)	1068 (25.5%)	615 (14.7%)
In your immediate family (yes)	7066 (51.9%)	3231 (61.3%)	2864 (68.5%)	971 (23.3%)
In caring for others (yes)	3224 (23.7%)	1689 (32.0%)	924 (22.1%)	611 (14.7%)
Chronic health complaints (yes)^f^	6896 (50.6%)	2778 (52.7%)	2223 (53.1%)	1895 (45.4%)

^a^Data are displayed as median, with the first and third quartile given within brackets

^b^Education was divided up in low (junior school), middle (comprehensive school) and high (college and university)

^c^Work status was categorized as employed (employee and self-employed), unemployed (consisting out of work for more than and less than 1 year), looking after others (e.g. a carer or parent), a student, retired and unable to work

^d^E.g. carer or parent

^e^Income was grouped as follows low (UK; less than £14,000, Italy and the Netherlands; less than €20,000), middle (UK; £14,000-£27,999, Italy and the Netherlands; €20,000-€39,999) and high (UK; more than £27,999, Italy and the Netherlands; more than €39,999)

^f^Chronic health complaints were defined as asthma, chronic bronchitis, severe heart disease, consequences of a stroke, diabetes, severe back complaints, arthrosis, rheumatism, cancer, memory problems due to neurological disease/dementia, memory problems due to ageing, depression or anxiety disorder, and other chronic health complaints

### DCE data

An upward trend was shown in probability of respondents choosing health state A when the difference in sum score of health state A and B (e.g. probability of choosing health state 11,111; sum score = 5 over health state 123,45; sum score = 15) becomes bigger and more positive, which is what was expected (Online Appendix B).

### VAS data

Table 3 shows the summary statistics for the three VAS health states considering the QOLIBRI-OS data. The lowest mean value was 38.01 (health state dead) and highest mean value was 81.49 (health state 11,111, e.g. very satisfied with every attribute). As expected, when the summary score of the of the health state decreased (e.g. severity of health state becomes lower), which means the health state was comprised of lower attribute levels, the utility mean increased.

**Table 3:** QOLIBRI-OS summary statistics for the 3 selected VAS health states

Health state	Observations (*N*)	Mean VAS	SD	Rescaled mean	Utility mean
Dead	116	38.01	40.71	0.00	0.00
55,555^a^	138	54.64	33.56	38.26	0.38
11,111^b^	245	81.49	22.15	100	1

^a^Worst possible health state; all attributes have ‘not at all satisfied’ level

^b^Best possible health state; all attributes have ‘very satisfied’ level

Note: rescaled mean for health state 55,555 per country UK (35.56), NL (26.07), Italy (49.97)

### Value sets

Table 4 shows the 20 parameters model per country for the QOLIBRI-OS which was based on the conditional logistic regression for the DCE data and the anchoring of the DCE coefficients. For all respondents and both the Netherlands and Italy, the lowest estimate for the DCE and anchored DCE data was found for ‘Quite satisfied with feelings and emotions’ and the highest estimate for ‘Not at all satisfied with how brain is working in terms of concentration, memory and thinking’. When looking at the model specifically for the UK, the lowest estimate was found for ‘Moderately satisfied with personal and social life’ and the highest for ‘Not at all satisfied with how brain is working in terms of concentration, memory and thinking’. The biggest increase in weight for all attributes is seen from level four (slightly satisfied) to level five (not at all satisfied). Table 5 introduces an example for the QOLIBRI-OS value set based on the DCE and anchored DCE models. This enables the calculation of a utility weight per health state, which is how the utilities for the QOLIBIR-OS data can be obtained. The utility scores of the anchored DCE model of the QOLIBRI-OS range from 0.383 for health state 55,555 to 1.0 for health state 11,111. Table 6 shows an example of values for a mild, moderate and severe health state. Generally speaking, Italians value these health states lower compared to their counterparts in UK and the Netherlands. Online Appendix C shows the non-parametric models per country for the QOLIBRI-OS and Online Appendix D shows an example of values for a mild, moderate and severe health state based on the non-parametric models.

**Table 4:** QOLIBRI-OS 20 parameters model per country

	All respondents			UK			The Netherlands			Italy		
	DCE data		Anchored DCE	DCE data		Anchored DCE	DCE data		Anchored DCE	DCE data		Anchored DCE
	*Estimate*	*SE*	*Estimate*^*1*^	*Estimate*	*SE*	*Estimate*^*1*^	*Estimate*	*SE*	*Estimate*^*1*^	*Estimate*	*SE*	*Estimate*^*1*^
Physical condition												
Quite	0.143	0.028***	0.015	0.171	0.048***	0.017	0.093	0.047*	0.010	0.158	0.051**	0.016
Moderately	0.313	0.028***	0.032	0.345	0.048***	0.035	0.311	0.047***	0.032	0.269	0.050***	0.028
Slightly	0.522	0.028***	0.053	0.500	0.049***	0.051	0.488	0.049***	0.050	0.568	0.051***	0.058
Not at all	1.306	0.031***	0.134	1.339	0.053***	0.137	1.080	0.053***	0.110	1.540	0.057***	0.157
How brain is working, in terms of concentration, memory and thinking												
Quite	0.200	0.027***	0.020	0.250	0.046***	0.026	0.105	0.047*	0.011	0.240	0.048***	0.025
Moderately	0.464	0.028***	0.047	0.426	0.047***	0.044	0.433	0.049***	0.044	0.542	0.051***	0.055
Slightly	0.663	0.025***	0.068	0.640	0.044***	0.065	0.609	0.043***	0.062	0.746	0.046***	0.076
Not at all	1.680	0.030***	0.172	1.688	0.052***	0.173	1.369	0.050***	0.140	2.033	0.054***	0.208
Feelings and emotions												
Quite	0.050	0.028	0.005	0.071	0.047	0.007	-0.013	0.048	-0.001	0.110	0.050*	0.011
Moderately	0.218	0.027***	0.022	0.159	0.046***	0.016	0.211	0.047***	0.022	0.328	0.050***	0.034
Slightly	0.290	0.026***	0.030	0.207	0.045***	0.021	0.416	0.045***	0.043	0.264	0.046***	0.027
Not at all	0.938	0.029***	0.096	0.967	0.049***	0.099	0.922	0.049***	0.094	0.969	0.052***	0.099
Ability to carry out day to day activities												
Quite	0.159	0.028***	0.016	0.136	0.049**	0.014	0.111	0.049*	0.011	0.232	0.051***	0.024
Moderately	0.306	0.028***	0.031	0.263	0.049***	0.027	0.259	0.047***	0.026	0.408	0.051***	0.042
Slightly	0.347	0.029***	0.035	0.268	0.050***	0.027	0.450	0.049***	0.046	0.356	0.052***	0.036
Not at all	1.131	0.028***	0.116	1.164	0.049**	0.119	1.143	0.049***	0.117	1.113	0.051***	0.114
Personal and social life												
Quite	0.109	0.028***	0.011	0.082	0.047	0.008	0.104	0.048*	0.011	0.121	0.050*	0.012
Moderately	0.225	0.031***	0.023	0.030	0.053	0.003	0.382	0.053***	0.039	0.252	0.056***	0.026
Slightly	0.421	0.028***	0.043	0.241	0.048***	0.025	0.698	0.049***	0.071	0.330	0.051***	0.034
Not at all	0.986	0.029***	0.101	0.833	0.050***	0.085	1.163	0.050***	0.119	0.963	0.052***	0.098
Constant/intercept	0.000		0.000	0.000		0.000	0.000		0.000	0.000		0.000

^a^β anchored DCE model = (β 20 parameter DCE model × (estimated VAS 55,555 / estimated DCE 55,555))

P-value: *** < 0.001. ** < 0.01. * < 0.05.. < 0.1

**Table 5:** QOLIBRI-OS example: the value for health state 21232

	DCE	Anchored DCE
Full health (constant/intercept)	1.000	1.000
Minus constant	0.000	0.000
Quite satisfied with physical condition	0.143	0.015
Very satisfied with how brain is working, in terms of concentration, memory and thinking	0.000	0.000
Quite satisfied with feelings and emotions	0.050	0.005
Moderately satisfied with ability to carry out day to day activities	0.306	0.031
Quite satisfied with personal and social life	0.109	0.011
Utility weight health state 21232	0.392^a^	0.938

Note: all respondents

^a^Calculation of utilities: utility = 1—value

**Table 6:** Example of values for a mild, moderate and severe health state

	Anchored DCE			
	All respondents	UK	The Netherlands	Italy
Best health state: 11,111	1.000	1.000	1.000	1.000
Mild health state: 21,232	0.902	0.940	0.955	0.918
Moderate health state: 34,343	0.631	0.853	0.799	0.801
Severe health state: 55,455	0.449	0.465	0.472	0.396
Worst health state: 55,555	0.383	0.383	0.383	0.383

*DCE* Discrete Choice Experiment

## Discussion

Our study demonstrates the first value sets for the QOLIBRI-OS. The main outcomes according to the preferences of our general sample suggested the biggest increase in weight was found when making the step from level slightly satisfied to level not at all satisfied within an attribute, which results in the largest impact on HRoQL. This is also in line with previous EQ-5D value set research [32]. Additionally, it was also found that ‘Not at all satisfied with how brain is working in terms of concentration, memory and thinking’ should receive the greatest weight in utility calculations in all three countries.

When looking at the face validity of the value set, it was shown that a lower level of satisfaction within a health state also corresponded to a lower utility.

Strengths of our study include the representativeness of the study sample and the large number of survey respondents included in our survey. A general population sample was used instead of a brain injury group, because then the benefit gained has been determined from a public perspective, who ultimately are the taxpayers and potential patients.

During this study, DCEs were used and as mentioned in previous research, this valuation technique has advantages in measuring health state valuations over methods such as standard gamble (SG) and time trade off (TTO) in terms of simplicity [35] and understandability. There are several methods of administration to conduct health state valuation studies, such as face-to-face using paper-and-pencil methods and web-based questionnaires. The choice for a web-based survey during this study also implied using health state valuation methods that were amenable to online administration, in this case DCEs and VAS. Compared to personal interviews, web-based surveys are equipped to get answers from large samples in a relatively short time, have a flexible sampling frame, enable a range of background characteristics of non-respondents to be obtained, the order of the questions can be randomized, allow complex routing of questions, the time it takes a respondent can be recorded, and the errors associated with data entry are minimized [36]. Some limitations considering DCE research are the fact that a main effects only design, assuming that all attributes were value-independent of each other (i.e. all interactions between attributes were zero) was used. This may, however, be reasonable since main effects typically account for 70–90% of the explained variance in DCE [37]. Additionally, the complexity of a DCE can potentially cause some extra selection bias compared with general questionnaire surveys [38]. Furthermore, we have also encountered some limitations specific to our study. To ultimately get to five items for the QOLIBRI-OS, we based eliminating the last item on subjective researcher judgement, which could potentially lead to bias. Considering it was a web-based survey, we had no control on checking if respondents completely understood the task at hand. For future studies it would be advisable to build in a tool, to be able to check answers while respondents are taking the survey, for example to check if they are using the VAS correctly and are not turning it upside down. Additionally, face-to-face surveys will deliver higher quality data, but also require larger monetary resources. However, web-based is the mostly used administration method in DCE research, especially because of the high costs associated with the face-to-face method. We based our health states and pairs on previous EQ-5D research; however, it could be that for the QOLIBRI-OS instrument different health states should have been asked and for future research it would be advised to develop an experimental design where the pairs of health states are selected specifically for the QOLIBIR-OS. Another problem is that the respondents saw a complete ‘clean’ VAS for every new question. In a situation where their given answers are shown on the scale during the following questions, the respondents can scale their own answers more easily, which ultimately leads to a better scale division. The VAS and DCE are different tasks; what people imagine when they use the VAS may vary relative to a DCE. Using the VAS to scale, such as was done in this study, makes mathematical sense, but does it also make sense when using it to scale coefficients giving utilities? In addition, the worst health state (health state 55,555, e.g. not at all satisfied with every attribute) was given a mean VAS value of 54.64, which was expected to be lower, and could influence the rescaling. Furthermore, VAS scores used for rescaling in this study were not based on country-specific data due to small sample size. Future research could solve these limitations linked to the VAS values used in this study by doing a small TTO valuation task in each of the three countries, to provide anchors for the DCE scale. In addition, for the UK value set, an inconsistent coefficient in the final algorithm (“moderately satisfied with personal and social life”) was reported, which should be looked at in more detail in future research. Moreover, the DCE and VAS questions were completely randomized. The quality of the data would have most likely been higher, if we asked the DCE and VAS questions in blocks, which would be randomly assigned to the respondents and every block consisted of one of the better health states and the worst health state [24]. This makes for a more balanced way of asking the questions, because everyone gets a well-balanced set of questions, which accounts for the whole range of severity. In addition, red-green colour blindness could have influenced our respondents while answering the DCE questions; however, color-coding does improve the results [39]. Building upon these findings, it would be recommended for future research to provide anchors for the DCE and to use different colours than red and green. Since we used a market research company to recruit our sample, some individuals might be ‘professional’ respondents: those who answer a large number of surveys, and whose responses are not typical for the general public and we do not know to what extent our samples are representative for the population in the three countries with regard to characteristics other than age, gender and educational level. Nonetheless, this study is the first one to determine a value set for the QOLIBRI-OS in three different European countries and introduced the opportunity to compare HRQoL of TBI patients with other (patient) groups. Similar studies have been performed for the EQ-5D [25, 32], and are used daily in HRQoL research.

## Conclusions

By transforming the QOLIBRI-OS into utility scores, we have enabled the potential application in economic evaluations and in summary measures of population health, which may inform decision-makers on the best interventions and strategies for TBI patients.

## Electronic supplementary material

Below is the link to the electronic supplementary material.

Supplementary file1 (DOCX 13 kb)

Supplementary file2 (TIF 163 kb)

Supplementary file3 (PDF 41 kb)

Supplementary file4 (DOCX 12 kb)
