# Supplementary material for: Health-related quality of life after traumatic brain injury: deriving value sets for the QOLIBRI-OS for Italy, The Netherlands and The United Kingdom
Source: Qual Life Res. 2020 Jul 15;29(11):3095–107. doi: 10.1007/s11136-020-02583-6 (PMC7591447; doi:10.1007/s11136-020-02583-6)
Supplement: Supplementary file 1 — Supplementary file1 (DOCX 13 kb) [file 11136_2020_2583_MOESM1_ESM.docx]

| **Appendix A.** Measures of Item Difficulty and Fit from Rasch Analysis of the QOLIBRI-OS | | | | | |
| --- | --- | --- | --- | --- | --- |
| **QOLIBRI-OS items** | **Item difficulty measure** | **Infit MNSQ** | **Infit Z** | **Outfit MNSQ** | **Outfit Z** |
| Physical condition | 0.32 | 1.05 | 0.9 | 1.07 | 1.4 |
| How brain is working, in terms of concentration, memory and thinking | 0.23 | 1.11 | 2.2 | 1.12 | 2.3 |
| Feelings and emotions | -0.01 | 1.04 | 0.8 | 1.02 | 0.5 |
| Ability to carry out day to day activities | -0.35 | 0.99 | -0.3 | 0.94 | -1.2 |
| Personal and social life | -0.38 | 1 | 0.1 | 0.93 | -1.4 |
| Current situation and future prospects | 0.19 | 0.81 | -4.1 | 0.8 | -4.4 |
| **Six items:** | | | | | |
| Person real separation: 1.57 | | | | | |
| Reliability: 0.71 | | | | | |
|  | | | | | |
| **Five items:** | | | | | |
| Person real separation: 1.63 | | | | | |
| Reliability: 0.73 | | | | | |
| * when the current situation and future prospects item has been excluded. | |  |  |  |  |
