# Supplementary figures and images for: Health-related quality of life after traumatic brain injury: deriving value sets for the QOLIBRI-OS for Italy, The Netherlands and The United Kingdom
Source: Qual Life Res. 2020 Jul 15;29(11):3095–107. doi: 10.1007/s11136-020-02583-6 (PMC7591447; doi:10.1007/s11136-020-02583-6)

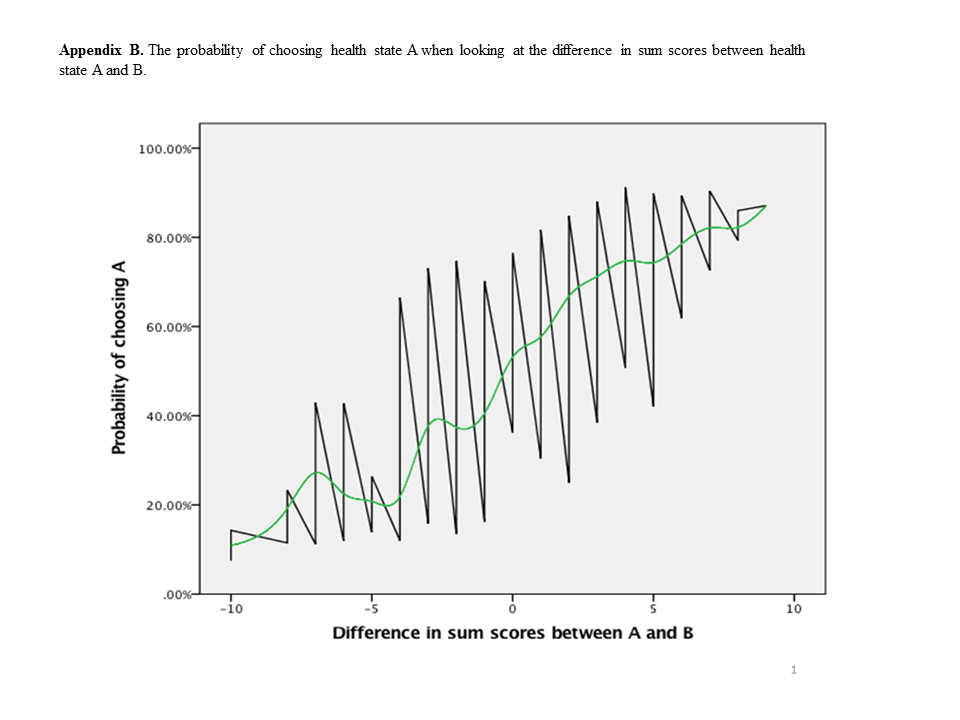

Supplement: Supplementary file 2 — Supplementary file2 (TIF 163 kb) [file 11136_2020_2583_MOESM2_ESM.tif]
