# Supplementary material for: Health-related quality of life after traumatic brain injury: deriving value sets for the QOLIBRI-OS for Italy, The Netherlands and The United Kingdom
Source: Qual Life Res. 2020 Jul 15;29(11):3095–107. doi: 10.1007/s11136-020-02583-6 (PMC7591447; doi:10.1007/s11136-020-02583-6)
Supplement: Supplementary file 3 — Supplementary file3 (PDF 41 kb) [file 11136_2020_2583_MOESM3_ESM.pdf]

Appendix C. QOLIBRI-OS non-parametric model per country

	**All respondents**				**UK**				**The Netherlands**				**Italy**			
	Unanchored		Anchored		Unanchored		Anchored		Unanchored		Anchored		Unanchored		Anchored	
	Mean	*95% CI*	*Mean*	*95% CI*	*Mean*	*95% CI*	*Mean*	*95% CI*	*Mean*	*95% CI*	*Mean*	*95% CI*	*Mean*	*95% CI*	*Mean*	*95% CI*
**Physical condition**																
Quite	0.121	0.057-0.182	0.012	0.006-0.019	0.135	0.029-0.241	0.014	0.003-0.025	0.090*	-0.017-0.195	0.010*	-0.002-0.021	0.128	0.017-0.240	0.012	0.002-0.023
Moderately	0.289	0.219-0.357	0.030	0.023-0.037	0.290	0.171-0.405	0.030	0.0178-0.042	0.317	0.211-0.422	0.034	0.023-0.045	0.260	0.135-0.375	0.025	0.013-0.036
Slightly	0.548	0.483-0.613	0.057	0.050-0.063	0.521	0.411-0.630	0.054	0.043-0.066	0.482	0.377-0.585	0.052	0.041-0.063	0.624	0.505-0.749	0.060	0.049-0.072
Not at all	1.349	1.275-1.421	0.139	0.132-0.146	1.419	1.290-1.549	0.148	0.1360.159	1.124	1.002-1.248	0.121	0.110-0.132	1.517	1.395-1.643	0.146	0.135-0.156
**How brain is working, in terms of concentration, memory and thinking**																
Quite	0.157	0.091-0.224	0.016	0.009-0.023	0.241	0.137-0.348	0.025	0.014-0.036	0.081*	-0.028-0.189	0.009*	-0.003-0.020	0.173	0.049-0.289	0.017	0.005-0.028
Moderately	0.380	0.308-0.451	0.039	0.032-0.046	0.376	0.259-0.488	0.039	0.027-0.051	0.353	0.228-0.476	0.038	0.025-0.051	0.459	0.336-0.579	0.044	0.032-0.055
Slightly	0.704	0.640-0.769	0.073	0.066-0.079	0.719	0.609-0.835	0.075	0.063-0.088	0.630	0.531-0.734	0.068	0.057-0.079	0.793	0.688-0.903	0.076	0.066-0.087
Not at all	1.619	1.548-1.691	0.167	0.160-0.173	1.597	1.486-1.716	0.167	0.156-0.177	1.354	1.239-1.476	0.146	0.135-0.157	1.959	1.836-2.086	0.188	0.177-0.199
**Feelings and emotions**																
Quite	0.166	0.100-0.235	0.017	0.010-0.024	0.253	0.141-0.369	0.026	0.015-0.038	-0.002*	-0.105-0.108	0.000*	-0.011-0.011	0.243	0.120-0.369	0.023	0.011-0.035
Moderately	0.223	0.159-0.288	0.023	0.017-0.030	0.168	0.062-0.275	0.018	0.007-0.029	0.225	0.117-0.336	0.024	0.013-0.036	0.301	0.185-0.419	0.029	0.018-0.040
Slightly	0.336	0.270-0.401	0.035	0.028-0.041	0.267	0.157-0.379	0.028	0.016-0.040	0.403	0.299-0.502	0.043	0.032-0.054	0.341	0.224-0.458	0.033	0.021-0.044
Not at all	0.940	0.877-1.005	0.097	0.091-0.103	0.936	0.835-1.045	0.098	0.089-0.107	0.911	0.807-1.020	0.098	0.088-0.108	1.013	0.896-1.135	0.097	0.088-0.107
**Ability to carry out day to day activities**																
Quite	0.132	0.058-0.205	0.014	0.006-0.021	0.127	0.007-0.249	0.013	0.001-0.026	0.077*	-0.046-0.197	0.008*	-0.005-0.021	0.223	0.100-0.346	0.021	0.010-0.033
Moderately	0.228	0.162-0.295	0.023	0.017-0.030	0.218	0.110-0.324	0.023	0.012-0.034	0.212	0.100-0.321	0.023	0.011-0.034	0.314	0.190-0.439	0.030	0.018-0.042
Slightly	0.330	0.261-0.404	0.034	0.027-0.041	0.236	0.124-0.354	0.025	0.013-0.037	0.440	0.327-0.557	0.047	0.035-0.060	0.343	0.210-0.489	0.033	0.020-0.046
Not at all	1.108	1.037-1.177	0.114	0.108-0.121	1.152	1.035-1.267	0.120	0.109-0.131	1.134	1.011-1.254	0.122	0.110-0.134	1.106	0.985-1.227	0.106	0.096-0.117
**Personal and social life**																
Quite	0.097	0.026-0.165	0.010	0.003-0.017	0.064*	-0.055-0.176	0.007*	-0.006-0.018	0.090*	-0.031-0.203	0.010*	-0.003-0.021	0.128	0.007-0.246	0.012	0.001-0.023
Moderately	0.251	0.174-0.330	0.026	0.018-0.034	0.025*	-0.1-0.156	0.003*	-0.010-0.016	0.376	0.254-0.494	0.040	0.028-0.053	0.348	0.198-0.497	0.033	0.019-0.047
Slightly	0.370	0.311-0.433	0.038	0.032-0.045	0.192	0.094-0.293	0.020	0.010-0.030	0.616	0.512-0.723	0.066	0.055-0.078	0.329	0.216-0.488	0.032	0.021-0.043
Not at all	0.989	0.925-1.051	0.102	0.096-0.108	0.915	0.801-1.026	0.095	0.085-0.106	1.140	1.036-1.245	0.123	0.113-0.133	0.923	0.811-1.033	0.089	0.079-0.098

* Not significant: p-value < 0.05

95% CI; 95% credible interval
