# Supplementary material for: Health-related quality of life after traumatic brain injury: deriving value sets for the QOLIBRI-OS for Italy, The Netherlands and The United Kingdom
Source: Qual Life Res. 2020 Jul 15;29(11):3095–107. doi: 10.1007/s11136-020-02583-6 (PMC7591447; doi:10.1007/s11136-020-02583-6)
Supplement: Supplementary file 4 — Supplementary file4 (DOCX 12 kb) [file 11136_2020_2583_MOESM4_ESM.docx]

| **Appendix D.** Example of values for a mild, moderate and severe health state | | | |  |
| --- | --- | --- | --- | --- |
|  | **All respondents** | **UK** | **The Netherlands** | **Italy** |
| Mild health state: 21232 | 0.950 | 0.948 | 0.960 | 0.942 |
| Moderate health state: 34343 | 0.833 | 0.879 | 0.789 | 0.827 |
| Severe health state: 55455 | 0.446 | 0.452 | 0.442 | 0.445 |
